# Ligation-assisted endoscopic enucleation for the diagnosis and resection of small gastrointestinal tumors originating from the muscularis propria: a preliminary study

**DOI:** 10.1186/1471-230X-13-88

**Published:** 2013-05-16

**Authors:** Jintao Guo, Zhijun Liu, Siyu Sun, Sheng Wang, Nan Ge, Xiang Liu, Guoxin Wang, Xianghong Yang

**Affiliations:** 1 Endoscopic Center, Shengjing Hospital of China Medical University, No. 36 Sanhao Street, Shenyang, Liaoning Province 110004, China; 2 Ultrasound Department, Shengjing Hospital of China Medical University, No. 36 Sanhao Street, Shenyang, Liaoning Province, 110004, China; 3 Pathological Department, Shengjing Hospital of China Medical University, No. 36 Sanhao Street, Shenyang, Liaoning Province, 110004, China

**Keywords:** Endoscopic resection, Ligation, Subepithelial tumor, Muscularis propria

## Abstract

**Background:**

Ligation-assisted endoscopic enucleation (EE-L) was developed for the pathological diagnosis and resection of small gastrointestinal tumors originating from the muscularis propria. The technique combines endoscopic band ligation and endoscopic enucleation. The aim of this study was to evaluate the efficacy and safety of EE-L in the diagnosis and resection of gastrointestinal tumors originating from the muscularis propria.

**Methods:**

A total of 43 patients were eligible for inclusion in this study from June 2009 to June 2011. Endoscopic ligation was first performed to force the tumor to assume a polypoid form with a pseudostalk. EE-L was then performed until the tumor was completely enucleated from the muscularis propria. Wound closure was performed using clips and adhesive tissue.

**Results:**

All 43 tumors were completely enucleated. The mean enucleation time was 7.2 minutes (range, 5–11 minutes). No perforation, massive hemorrhage, or peritonitis requiring further endoscopic or surgical intervention occurred. Histopathology, 19 lesions were identified as gastrointestinal stromal tumors and 24 lesions were identified as leiomyomas. The mean follow-up time was 20.4 months (range, 14–38 months). No recurrence has occurred during the follow-up period.

**Conclusions:**

EE-L appears to be a safe, effective, and relatively simple method for the histologic diagnosis and removal of small gastrointestinal tumors originating from the muscularis propria.

## Background

Some gastrointestinal tumors originating from the muscularis propria, such as gastrointestinal stromal tumors (GISTs), may be nonmalignant when diagnosed but have the potential to undergo malignant transformation. A majority of patients with gastrointestinal lesions originating from the muscularis propria prefer to undergo resection despite controversies over therapeutic decisions. Several endoscopic resection techniques have been proven feasible and safe for tumors originating from the muscularis propria, including endoscopic submucosal dissection [1-4], endoscopic enucleation [5,6], endoscopic ligation [7-9], endoscopic ligation and resection [10], endoscopic full-thickness resection [11], and submucosal tunneling endoscopic resection [12,13].

Ligation-assisted endoscopic enucleation (EE-L) was developed by combining endoscopic band ligation and endoscopic enucleation to fully exploit the advantages of each technique. The present study investigated the efficacy and safety of EE-L in the diagnosis and resection of gastrointestinal tumors originating from the muscularis propria.

## Methods

### Patients

Patients who underwent EE-L for gastrointestinal tumors originating from the muscularis propria at Shengjing Hospital of China Medical University from June 2009 to June 2011 were enrolled in this study. To be included in the study, the tumors had to originate in the muscularis propria layer of the gastrointestinal wall, and this had to be confirmed by endoscopic ultrasonography (EUS). All tumors eligible for participation based on EUS examination were no more than 10 mm in diameter because the diameter of the air-driven ligator cap was 10 mm. All patients in this series had a normal complete blood cell count and thrombin time without having taken warfarin, clopidogrel, aspirin, or any other nonsteroidal anti-inflammatory drug for at least 1 week before the procedure.

This study was approved by the Institutional Review Board and Ethics Committee of China Medical University. All patients voluntarily chose their therapeutic course and provided written informed consent for their participation in this study. The operator performing the EE-L procedure in this study was familiar with both the endoscopic ligation and submucosal dissection techniques.

### Devices

Endoscopic ultrasound was performed with a linear-array scanning echoendoscope (Pentax EG3870UT equipped with a HITACHI 6500 EUB ultrasonography machine) or a radical scanning echoendoscope (SP-701; Fujinon). Endoscopic ligation was performed with a standard endoscope (EPK-I; Pentax) with a 10-mm air-driven ligator cap (Sumibe, Akita, Japan). This ligator cap had a small tube to control the band, which was released after 2 ml of air had been injected into the tube. EE-L was performed using equipment including a hook knife (KD-620LR; Olympus), injection needle (NM-4L-1; Olympus), forceps, snare (SD-9L-1; Olympus), hemostatic forceps (FD-410LR; Olympus), and high-frequency generator (ICC 200; Erbe, Tübingen, Germany). Wound closure was performed with endoclips (HX-600-135; Olympus) and tissue adhesive composed mainly of alkyl alpha-cyanoacrylate (Beijing Suncon Medical Adhesive Co., Beijing, China). About 1.5 to 3.0 ml of adhesive was sprayed evenly over the wound by endoscopic catheters that were placed in the stomach and aimed at the wound surface.

### Procedure

**Figure 1:**
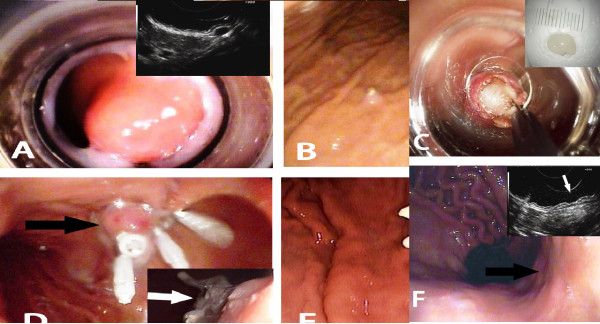
**EE-L for gastric tumor originating from muscularis propria layer. A **Images of tumor prior to banding. **B **Image of tumor ligated by an elastic band. **C** Images of tumor after exposure and after resection (inset). **D **The wound surface was closed with metallic clips (black arrow) and then covered by tissue adhesive (white arrow, inset). **E **Wound surface 5 days after EE-L showing the wound surface covered with the elastic band, metallic clips, and tissue adhesive (black arrow); wound surface 1 month after EE-L showing the iatrogenic ulcer (white arrow, inset). **F** Wound surface 2 months after EE-L showing the scar. Endoscopic view (black arrow) and EUS view (white arrow, inset).

**Figure 2:**
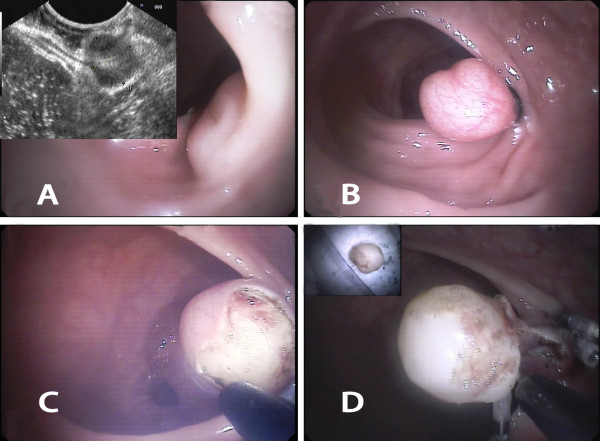
**EE-L for rectal tumor originating from the muscularis propria layer. A **Images of rectum tumor prior to banding. **B **Image of tumor ligated by an elastic band. **C** Image of exposed tumor. **D **Images of tumor completely enucleated and then resected (inset).

The lesion was first aspirated into the transparent cap attached to the tip of endoscope. The elastic band was then released around its base (Figures 1A, B; 2A, B). The purpose of ligation was to force the lesion to assume a polypoid form with a pseudostalk. EUS was used to determine whether the hypoechoic mass had been completely confined within the band. If the lesion was not completely ligated, the band was removed with a foreign body forceps and the lesion was ligated again. After the mucosal and submucosal layers overlying the tumor had been cut open with hook knife, the exposed tumor was gradually dissected from the muscularis propria layer by the hook knife and\or forceps (Figures 1C, D; 2C-E). When the tumor had been completely exposed, an electrocautery snare was used in some cases for the last step of the excision. After the wound had been carefully evaluated to ensure the absence of residual tumor tissue, the wound was closed with metallic clips and tissue adhesive was sprayed on the clips (Figure 1E, F). The patients received proton pump inhibitors until the iatrogenic ulcers had completely healed.

Pathologic examination included identification of cell type, overall cellularity, nuclear atypia, immunohistochemical findings, and the mitotic index. Immunohistochemical analysis for CD117 (c-kit), CD34, smooth muscle actin, desmin, S-100, etc. were performed to differentiate tumors of mesenchymal origin.

Endoscopic examinations were performed 5 days after the procedure to examine the wound surface and again 2 months after the resection to assess ulcer healing. To confirm the completeness of tumor resection, endoscopic examinations were performed for all patients once a year for the first 2 years. If no residual tumor or tumor recurrence was found, the patients diagnosed with leiomyomas required no further treatment; the patients diagnosed with GISTs were advised to undergo endoscopic examinations once every 2 years. If residual tumor or tumor recurrence was detected, EE-L could be performed.

## Results

From June 2009 to June 2011, a total of 43 patients with 43 tumors underwent EE-L at Shengjing Hospital of China Medical University. Patient demographic characteristics, lesion features, pathological diagnosis, and clinical outcomes are summarized in Table 1. None of the patients included in the study had significant symptoms.

**Table 1:** Demographic and clinicopathological characteristics of the study patients (N = 43)

**Case no.**	**Gender/age (y)**	**Location**	**Tumor size (mm)**	**Enucleation time (min)**	**Operation time (min)**	**Pathological diagnosis**	**Complications**
1	F/45	Fundus, PW	9 × 6	6	30	Leiomyoma	None
2	M/48	Body, LC	8 × 7	7	33	GIST	None
3	M/37	Body, PW	10 × 4	7	38	Leiomyoma	None
4	F/65	Cardia	9 × 5	5	39	Leiomyoma	None
5	M/38	Body, AW	10 × 7	10	40	Leiomyoma	None
6	F/56	Fundus, PW	9 × 8	5	25	GIST	None
7	M/44	Fundus, PW	9 × 8	6	34	GIST	None
8	F/51	Body, LC	10 × 6	8	29	Leiomyoma	None
9	F/50	Body, LC	10 × 8	9	25	GIST	None
10	M/60	Fundus, PW	8 × 6	10	30	Leiomyoma	None
11	M/39	Body, PW	6 × 5	11	32	GIST	None
12	F/43	Body, PW	9 × 5	8	32	Leiomyoma	None
13	F/31	Cardia	9 × 8	7	26	GIST	None
14	M/56	Fundus, PW	10 × 7	5	30	GIST	None
15	M/47	Body, GC	5 × 4	8	28	Leiomyoma	None
16	F/48	Cardia	10 × 8	9	27	GIST	Bleeding
17	M/53	Fundus, PW	9 × 7	10	32	Leiomyoma	None
18	M/55	Fundus, PW	7 × 6	11	28	Leiomyoma	None
19	F/68	Body, PW	8 × 5	5	27	Leiomyoma	None
20	M/59	Rectum	9 × 8	5	23	GIST	None
21	F/27	Body, LC	9 × 4	7	26	Leiomyoma	None
22	M/36	Fundus, PW	10 × 8	8	22	GIST	None
23	F/54	Body, PW	8 × 5	9	29	Leiomyoma	None
24	M/32	Cardia	10 × 9	6	25	GIST	None
25	M/35	Body, PW	9 × 6	5	20	Leiomyoma	None
26	F/53	Antrum, PW	8 × 7	7	23	GIST	None
27	M/29	Body, LC	10 × 7	8	27	Leiomyoma	None
28	M/46	Fundus, GC	9 × 5	10	32	Leiomyoma	None
29	F/49	Fundus, PW	10 × 5	11	29	Leiomyoma	None
30	M/36	Body, AW	9 × 8	8	27	GIST	None
31	F/47	Fundus, PW	8 × 7	6	24	GIST	None
32	F/48	Cardia	9 × 6	5	19	Leiomyoma	None
33	M/52	Body, PW	10 × 6	7	21	Leiomyoma	None
34	F/51	Body, LC	9 × 8	5	32	GIST	None
35	M/47	Rectum	10 × 9	6	32	Leiomyoma	None
36	M/36	Fundus, PW	9 × 5	5	26	Leiomyoma	None
37	F/53	Fundus, PW	10 × 8	7	30	GIST	None
38	M/49	Body, PW	10 × 8	8	32	GIST	None
39	F/43	Body, LC	8 × 4	5	32	Leiomyoma	None
40	F/28	Cardia	9 × 7	5	25	GIST	None
41	F/48	Body, PW	10 × 6	6	16	Leiomyoma	None
42	M/37	Cardia	8 × 7	6	19	Leiomyoma	None
43	F/49	Fundus, PW	7 × 6	7	23	GIST	None

*GIST*, gastrointestinal stromal tumor; *PW*, posterior wall; *AW*, anterior wall; *LC*, lesser curvature; *GC*, greater curvature.

Note: We have received consent from each patient to publish this specific information.

All 43 gastrointestinal tumors were resected by EE-L. The mean operation time was 27.9 minutes (range, 16–40 minutes), and the mean enucleation time was 7.2 minutes (range, 5–11 minutes). One patient experienced self-limiting, non-life-threatening hemorrhage 5 days after EE-L. No perforation, massive hemorrhage, or peritonitis requiring further endoscopic or surgical intervention occurred. The mean follow-up time was 20.4 months (range, 14–38 months). No recurrence has occurred during the follow-up period.

The histological diagnoses obtained for the 43 lesions were 24 leiomyomas and 19 GISTs. Mitotic counts in all 19 GISTs were <5 per 50 high-power fields; thus, all were classified as very low-risk.

## Discussion

Gastrointestinal tumors of muscularis propria origin include leiomyomas, GISTs, neural tumors, and others. GISTs are the most common mesenchymal tumors of the gastrointestinal tract. Large GISTs with high mitotic rates are often associated with malignant behavior and display higher rates of recurrence and metastasis [14-18]. Therefore, the presence of such lesions can become a psychological burden to patients, even if the lesions are very small. For these reasons, some patients with such lesions prefer to undergo resection, although the management of small, incidentally discovered GISTs of <2 cm is controversial [19,20]. A histologic diagnosis of these lesions is sometimes needed [19-21]. EUS-guided sampling, deep biopsy, and endoscopic partial resection with the unroofing technique are feasible and effective procedures with which to obtain a definitive pathological diagnosis of subepithelial tumors originating from the muscularis propria [22-26]. However, these biopsy procedures are difficult for small tumors [19,20], especially those smaller than 10 mm. The true malignant potential for individual GISTs cannot be accurately determined using current imaging and noninvasive sampling methods [27].

Laparoscopic resection appears to be a safe and effective alternative method for the treatment of gastric tumors [28]. However, its application to small tumors is limited, especially those less than 10 mm. Several endoscopic resection techniques have potential advantages for small lesions originating from the muscularis propria. Endoscopic band ligation is a simple, effective, and safe procedure for treating gastrointestinal submucosal tumors of less than 10 mm, although it does not allow for a complete pathological examination because the tumor mass is exfoliated directly into the lumen and excreted [7,8]. Endoscopic enucleation or endoscopic submucosal dissection using an insulated-tip knife, hook knife, forceps, or electrocautery snare may have the advantage of providing both a pathological diagnosis and clinical treatment for such lesions in selected patients [1-6]. However, these techniques usually require highly skillful manipulation by experienced specialists and relatively longer procedure times [1-6,29]. In one study, the complete resection rate for small tumors, especially those smaller than 1 cm, was lower than that for larger tumors [5]. It was more difficult to strip the covering mucosa and dissect the submucosal layer in the small tumors [5].

Ligation-assisted endoscopic mucosal resection is one of several endoscopic treatment modalities employed in the treatment of esophageal neoplasia [30]. Endoscopic submucosal resection with a ligation device was successfully performed in all 25 esophageal subepithelial tumors localized within the muscularis mucosa or submucosa by Lee et al. [31], and the en bloc resection rate was 100% (25 of 25 tumors). Endoscopic ligation and resection shows promise as a safe and feasible technique with which to treat small EUS-suspected gastric GISTs [10].

EE-L was developed for the diagnosis and treatment of small gastrointestinal lesions originating from the muscularis propria. This technique combines endoscopic band ligation and endoscopic enucleation to fully exploit the advantages of each technique.

Endoscopic band ligation of the tumor simplifies the endoscopic enucleation procedure and reduces the time required because the elastic bands firmly ligate the lesions and cause them to assume a polypoid form with pseudostalks during the entire enucleation process. After the tumor has been ligated, the overall endoscopic enucleation process progresses easily and smoothly. The enucleation time in the present study was short (mean, 7.2 minutes; range, 5–11 minutes).

Perforation is a recognized complication during endoscopic resection, even in the hands of an expert endoscopist [1-6]. The EE-L technique may substantially decrease the risk of perforation during the enucleation process. First, the base below the tumor is firmly ligated by the elastic band. Second, precutting the overlying mucosa and submucosa above the tumor and then gradually enucleating the tumor maintains the major overlying mucosa, facilitating the wound closure procedure and promoting wound healing [2]. Third, closing the wound with clips and tissue adhesives prevents perforation and promotes wound healing. Clips have been widely applied for wound closure of the gastrointestinal tract to prevent perforation and bleeding [32-35].

Tissue adhesives are used for a variety of local applications including hemostasis, wound closure, and fistula repair. The most commonly utilized tissue adhesives in gastrointestinal endoscopy include cyanoacrylates, fibrin glues, and thrombin. Cyanoacrylates are widely used outside of the United States for gastric variceal bleeding and, to a lesser extent, ulcer bleeding and fistula closure [36]. Tissue adhesives sprayed onto the surface of the clips and wound firmly immobilize the clips onto the wound. This not only effectively prevents perforation, but also prevents bleeding from the iatrogenic ulcers [37,38].

This study has some limitations. Like other endoscopic resection techniques for removal of tumors arising from the muscularis propria, EE-L is limited in that complete enucleation is defined solely by endoscopic observation. It was impossible to remove tumor tissue with a sufficiently safe margin using endoscopic resection. Therefore, long-term follow-up assessment should be performed to ensure the complete removal of GISTs. Although endoscopic resection cannot completely eliminate the need for continued surveillance in all patients, it can eliminate the need for surveillance in patients with leiomyomas. For patients with GISTs in this study, endoscopic resection achieved complete tumor resection based on gross observation, and no residual tumor or tumor recurrence was found during the long-term follow-up.

## Conclusions

This study shows that EE-L appears to be a safe, effective, and relatively simple technique for the pathological diagnosis and resection of small gastrointestinal tumors originating from the muscularis propria. Controlled clinical trials with larger sample sizes and longer follow-up periods are necessary to further examine the value and limitations of this technique.

## Abbreviations

EE-L: Ligation-assisted endoscopic enucleation; GIST: Gastrointestinal stromal tumor; EUS: Endoscopic ultrasonography.

## Competing interests

JG, SS, ZL, SW, NG, XL, GW, and XY declare that they have no competing interests.

## Authors’ contributions

SS carried out the study design, obtained funding, and performed the endoscopic and EE-L procedures. GJ participated in the study design and drafting of the manuscript. LZ participated in the study design and helped to draft and critically revise the manuscript. WS and GN helped with performance of the EE-L procedure and clinical management of the patients. LX carried out the data acquisition, performed the statistical analysis, and helped with clinical management of the patients. WG was involved in data acquisition and clinical management of the patients. YX participated in the study design, carried out the pathological diagnosis, and participated in data acquisition. All authors read and approved the final manuscript.

## Pre-publication history

The pre-publication history for this paper can be accessed here:

http://www.biomedcentral.com/1471-230X/13/88/prepub
